# Our Journal

**Published:** 1846-04

**Authors:** 


### PART IV.—EDITORIAL DEPARTMENT.

### ARTICLE VII.

We present our Journal, to our readers, in the present No., in a new form, much enlarged, and, we hope, in every respect, more worthy of their patronage. We have long felt that our space was too contracted to allow us to do justice, either to the advance of Medical Science, or to our readers. At length, in accordance with the expressed wish of a large number of our subscribers, and our own long cherished desire, we feel warranted in extending our limits threefold; and that our contributors may not experience the necessity of condensing into a compass, too small to do justice to their subject, the communications with which we hope to be favored by them, we have thrown two *monthly* numbers into one *bimonthly*, each containing 96 pages. This will afford us ample space for the discussion of all subjects of interest that may present themselves. As a number of our subscribers and anticipated contributors are residents of the State of Indiana, in which there has, as yet, no medical journal been issued, and the services of Dr. John Evans having been secured as cöeditor, we have altered the title of our journal to express the better its more extended sphere, and will, hereafter, issue it simultaneously at Chicago, Illinois, and Indianapolis, Indiana. Our labor also, will be further divided by the association of *four* cöeditors, as seen by our title page. By this division of labor we hope to improve the Journal in spirit and practical value. Additionally, a number of accomplished medical gentlemen in our own and neighboring States and Territories, have kindly acceded to our request to become regular contributors to our pages. By this combination of effort we hope to make our Journal adequate to the wants of the North-Western section of the United States, in which region we have been the first, and as yet, the only exponent of the profession.

